# Medication Literacy in a Cohort of Chinese Patients Discharged With Essential Hypertension

**DOI:** 10.3389/fpubh.2019.00385

**Published:** 2020-01-09

**Authors:** Zhuqing Zhong, Guiyue Ma, Feng Zheng, Yinglong Duan, Siqing Ding, Aijing Luo

**Affiliations:** ^1^Third Xiangya Hospital, Central South University, Changsha, China; ^2^Key Laboratory of Medical Information Research, Central South University, College of Hunan Province, Changsha, China; ^3^Xiangya School of Nursing, Central South University, Changsha, China

**Keywords:** essential hypertension, discharged patients, medication literacy, medication compliance, antihypertensive drug

## Abstract

**Background:** In recent years, research on medication literacy has increased in many countries. Medication literacy in patients with essential hypertension affects the management and prognosis of hypertension.

**Method:** This is a cross-sectional study of 147 discharged patients with essential hypertension who were treated at a tertiary hospital in Changsha, Hunan, China, between March and June 2016. The demographic and clinical data of the patients with hypertension were obtained from the medical records. The Chinese version of the Medication Literacy Questionnaire was applied to measure the medication literacy of hypertensive patients from 7 to 30 days after discharge by structured interview. Data were analyzed using SPSS version 19.0. Multiple linear regression was used to analyse the meaningful determinants of medication literacy.

**Results:** The medication literacy of discharged patients with hypertension was poor. More than 70% of patients had no substantial knowledge of the effects and side effects of the medications they were taking, more than 30% of patients did not know the name or dose of the medication, and more than 20% of patients did not know how often to take the medication.

**Conclusion:** It is necessary to conduct targeted health education for discharged patients with essential hypertension to reduce the risks of low medication literacy based on the determinants obtained in this study.

## Introduction

Medication literacy refers to the ability of individuals to obtain, correctly understand and use medication information in order to take that medication safely and appropriately (1). Medication literacy can be used as a significant predictor of the accuracy of medication use behavior (2). It is critical for patients with hypertension to use medicine regularly and correctly over the long term to reduce the occurrence of major cardiovascular events (3). However, medication use among patients with hypertension is often inappropriate or problematic (4). Therefore, patients with hypertension have a higher risk of adverse drug events and readmission (5).

According to the Annual Report on Cardiovascular Diseases in China in 2015, there were 270 million patients with hypertension in the country (4). The prevalence of hypertension in China is 65%, with an annual cost of 40 billion yuan (6). Among the reasons for the high prevalence of hypertension in China, poor medication adherence is predominant. In the absence of proper medical supervision, self-treatment, and self-medication is a common phenomenon in patients with hypertension (7). More importantly, these patients often lack the knowledge to use antihypertensive drugs appropriately and safely. Therefore, the safety of self-medication of patients with hypertension has become a major concern in China.

Chinese scholars have investigated patients with hypertensive stroke after discharge and found that their medication adherence was poor. Additionally, the results showed that patients had a lack of knowledge of hypertension (8). In addition, studies have shown that low medication adherence is associated with a higher rate of hospitalization and higher mortality in patients with cardiovascular disease (9). Moreover, the inappropriate use of antihypertensive drugs is very high among outpatients with essential hypertension. Peng et al. (10) investigated the medication adherence of 102 discharged hypertensive patients, and the results showed that 68 patients had poor medication adherence, mainly due to the lack of knowledge of hypertension and poor cognitive ability. Thus, we conducted a cross-sectional study of medication literacy among patients with essential hypertension discharged from hospital in Changsha, Hunan, China. We aimed to investigate the level of medication literacy in a cohort of Chinese patients with essential hypertension discharged from the hospital and the meaningful determinants of medication literacy.

## Materials and Methods

### Study Setting

This study was conducted in patients with essential hypertension who were treated at the Third Xiangya Hospital of Central South University, Changsha, Hunan, China, from March to June 2016.

### Study Design

A cross-sectional study of all discharged hypertensive patients was conducted using a structured interview.

### Participants

The participants were patients diagnosed with essential hypertension who were treated at the Third Xiangya Hospital of Central South University in Changsha, Hunan, China from March to June 2016. The inclusion criteria were (1) age ≤ 85 years and free of language communication barriers; (2) taking antihypertensive drugs ≥ 2 weeks; and (3) no history of mental illness. The exclusion criteria were (1) mental instability or severe mental disorders; and (2) major chronic diseases such as chronic obstructive emphysema or severe hepatic or renal insufficiency. This study was approved by the Research Ethics Board of the Third Xiangya Hospital of Central South University (project number: 2016-s001). All participants were provided written informed consent.

### Study Variables

The level of medication literacy was the dependent variable and the sociodemographic characteristics (age, gender, education level, marital status, number of medications at discharge, length of hospital stay) were the independent variables.

### Data Collection

On the day of discharge, the demographic and clinical data, such as age, gender, education level, medication status and length of hospital stay, of the hypertensive patients were obtained from the medical records. Within 7 and 30 days of discharge, team members called the patients to assess their medication literacy based on an outlined structured interview. Medication literacy was measured by the Chinese version of the Medication Literacy Questionnaire for Discharged Patients developed by Maniaci et al. (11) of Mayo Clinic in the United States. This questionnaire has been translated into Chinese, and modifications have been made according to Chinese culture (2). The questionnaire was designed to assess the patient's ability to understand, calculate, and process drug information. The questionnaire contains 9 items, which are graded by a dichotomy system: one point for a correct answer and zero points for a wrong answer. Upon discharge, the attending physician provides the patient with a description of the prescribed drug, including its name, dose, frequency of use, therapeutic effect, and major side effects. The participants' answers were compared with the instructions from the medical staff. The answer to item 7 is only “yes” or “no,” and the answer to item 9 is some specific names. Thus, items 7 and 9 do not contribute to the total score. Therefore, the total score of this questionnaire is 7 points. A higher score indicates a higher level of medication literacy. For a single patient, the possible score is 0 to 7. Cronbach's α coefficient of the Chinese version of the questionnaire content was 0.85, the validity index was 0.81, and the test-retest reliability coefficient was 0.94 (2).

### Data Analyses

Demographic information was analyzed by descriptive statistical analysis. The patient's baseline characteristics and level of medication literacy were described by means, standard deviations (SD) and percentages. *T*-tests were used for univariate analysis, and determinants of medication literacy were analyzed by multiple linear regression. All determinants considered in this study were incorporated into the multiple linear regression model. Two-tailed tests of significance with *p* < 0.05 were used to establish statistical significance. All data analyses were performed using SPSS version 19.0 (2010, IBM Corp., Armonk, New York, United States).

## Results

### Sociodemographic Characteristics of the Participants

Before data collection, the sample size was calculated to ensure the statistical power of the study. A total of 147 patients with essential hypertension who were discharged from the hospital were enrolled in this study. Five patients refused to participate and 10 patients did not complete the questionnaire. Finally, 132 patients (91.0%) were analyzed in this study. Among the 132 patients, 70 (53.0%) were female and 62 (47.0%) were male. The mean age (SD) of the patients was 59.4 (15.7) years old, the mean number of medications at discharge was 4.4 (2.2), and the mean number of days in the hospital was 8.1 (3.7) days. Moreover, the patients had been diagnosed with essential hypertension for an average of 14 years.

### Medication Literacy

The medication literacy of the patients with essential hypertension who were discharged from hospital is shown in Table 1. Overall, the mean score for medication literacy (SD) was 4.89 (1.28). Even though all patients knew that they needed to take the medication after discharge, only 55.3% of patients knew the daily dosage they ought to take, 43.9% knew the name of the medication they were taking, and only 28% of patients knew the effects and adverse reactions of the medication they were taking.

**Table 1:** Medication literacy of patients with essential hypertension discharged from the hospital, Changsha, Hunan province, China, March to June 2016 (*n* = 132).

**Items**	**Number of correct answers**	**Proportion (%)**
1. Have you taken any medicine since you left the hospital?	132	100.0
2. How many medicines do you need to take every day?	73	55.3
3. Do you know the names of the medicines you are taking?	58	43.9
4. Do you know the dose of the medicine you are taking?	87	65.9
5. Do you know how often you should take the medicines?	131	99.2
6. Do you know the effects of each medicine you are taking?	128	97.0
7. Have you ever been warned about the side effects of the medicine you are taking?	22	16.7
8. Do you know the side effects of the medicines you are taking?	37	28.0
9. Do you know whom you should ask about the medicines you are taking?		
Local doctors	32	24.2
Doctors who write the prescription	51	38.6
Pharmacist	6	4.5
I don't know	35	26.5
Others	8	6.1

### Determinants of Medication Literacy

Table 2 shows the results of the univariate analysis. Three factors were strongly associated with medication literacy, with higher scores found among younger, highly educated patients, with a longer hospital stay.

**Table 2:** Results of univariate analysis of determinants of medication literacy for patients with essential hypertension discharged from the hospital, Changsha, Hunan, China, March to June 2016 (*n* = 132).

**Variables**	**Mean (SD) of total score**	***T*/*H***	***p***
Age		4.078	0.000
<65years	5.23 ± 1.30		
≥65 years	4.35 ± 1.05		
Gender		0.759	0.449
Male	4.81 ± 1.27		
Female	5.60 ± 0.97		
Year of schooling		7.550	0.000
≤6 years	4.08 ± 0.97		
7–9	4.81 ± 1.24		
10–12	4.98 ± 1.26		
>12	5.76 ± 1.18		
Marital status		2.526	0.084
Married	4.93 ± 1.29		
Unmarried	4.30 ± 0.95		
Number of medicines taken at discharge		−0.257	0.798
≤7	4.87 ± 1.29		
>7	5.00 ± 1.22		
Length of hospital stay		−2.153	0.033
≤8 days	4.72 ± 1.26		
>8 days	5.22 ± 1.26		

*T, Two-sample t-test; H, Kruskal-wallis H*.

Table 3 shows the results of the multiple linear regression analysis. Three factors were independently correlated with medication literacy. Medication literacy scores increased with the educational level and length of hospital stay but decreased with age.

**Table 3:** Results of multiple linear regression analysis of determinants of medication literacy for patients with essential hypertension discharged from hospital, Changsha, Hunan, China, March to June 2016 (*n* = 132).

**Determinants**	***B***	***SE***	***p***
Age (every 10 years)	−0.022	0.009	0.018
Education (each year of schooling)	0.428	0.159	0.008
Hospital stay (each day of stay)	0.059	0.027	0.030

*B, partial regression coefficient; SE, standard error*.

## Discussion

In this study, the medication literacy of a cohort of patients with essential hypertension discharged from the hospital was found to be insufficient. More than 70% of patients had no substantial knowledge of the effects and side effects of the medications they were taking, more than 30% of patients did not know the name or dose of the medication, and more than 20% of patients did not know how often to take the medication. Medication literacy can improve patients' compliance with drugs, thereby improving health outcomes. Therefore, it is crucial to improve the medication literacy of these patients in China. Our study also found that age, educational level, and length of hospital stay were independently associated with medication literacy. In our study sample, the level of medication literacy decreased with age. This result is consistent with the findings of previous studies (12–14). Older patients show poorer cognitive abilities, and therefore their learning and memory abilities may be not as good as younger patients.

The results indicated that high education level was associated with a better level of medication literacy. This finding is inconsistent with that of Maniaci et al. (11) who found no relationship between years of education and medication literacy. The level of education was positively correlated with medication literacy, indicating that general literacy and knowledge may improve the understanding of medication information and health issues and therefore be conducive for improving medication literacy. The multiple linear regression results showed that the length of hospital stay was an independent factor effecting medication literacy. The positive association between length of hospital stay and the level of medication literacy is unique and is reported here for the first time. We speculate that the longer the patients stayed in the hospital, the more health information they received from doctors and other patients, including information concerning essential hypertension and antihypertensive drugs. In addition, these patients may pay more attention to their health conditions while in hospital, thus improving their medication literacy. Furthermore, studies have shown that there is a positive correlation between age and length of hospital stay (15), but our study did not draw similar conclusions.

A lack of medication literacy increases the risk of re-hospitalization and emergency department visits in patients with essential hypertension. Additionally, serious adverse events may be related to unsafe medication use (16, 17). Effective communication on safe medication use between patients and health care professionals is important for improving medication literacy and reducing medication use errors (12, 18). Providing patients with comprehensive written medication education materials is another important method for reducing confusion, as medication information is often forgotten when communicated orally (19). Meanwhile, follow-up after discharge could remind patients to pay more attention to their medication use.

## Strengths and Limitations of the Study

Our study has several strengths. First, although studies on medication literacy for patients with essential hypertension discharged from the hospital have been conducted in other populations, to the best of our knowledge, this is the first study that has measured the medication literacy and examined the determinants of medication literacy in patients with essential hypertension discharged from the hospital in the Chinese population. China's population needs to be studied because of significant differences in its culture and health care system compared to other populations or jurisdictions. Second, we used a validated tool to measure medication knowledge, which contributed to the reliability and validity of the results. Third, all patients investigated in this study had homogeneous diseases. The accurate diagnosis of hypertension in a tertiary care center in China further strengthened the results of this study. Fourth, the collected data were analyzed using reliable statistical methods, and the results were easy to interpret. The limitations of our study should be recognized. First, the study comprised only a small number of participants from one hospital in China. Thus, these results may not be applicable in other regions. Further research is needed to increase the sample size and extend the finding to populations in other regions of China. Second, the same tool was used to access participants of different ages, but this tool may not be appropriate for different age groups.

## Conclusion

Medication literacy is insufficient for patients with essential hypertension discharged from the hospital. Age, educational level and length of hospital stay are important determinants of medication literacy. Medication literacy decreases with age, but increases with educational level and length of hospital stay. It is necessary to conduct health education for all patients with essential hypertension discharged from hospital to reduce the risk of low medication literacy.

## Data Availability Statement

The datasets generated for this study are available on request to the corresponding author.

## Ethics Statement

This study was carried out in accordance with the Chinese recommendations for essential hypertension and the Declaration of Helsinki, and written informed consent was obtained from all of the subjects. The protocol was approved by the Research Ethics Board of the Third Xiangya Hospital of Central South University.

## Author Contributions

ZZ designed the study, participated in the data processing and statistical analysis, and wrote the initial draft of the manuscript. GM, FZ, and YD participated in the design of the study and questionnaire administration and discussed the analytical results. SD provided important feedback on the manuscript. AL participated in the design of the study and provided important feedback on the manuscript. All authors read and approved the final manuscript.

### Conflict of Interest

The authors declare that the research was conducted in the absence of any commercial or financial relationships that could be construed as a potential conflict of interest.

## References

[1] Pouliot A Vaillancourt Régis Stacey D Suter P. Defining and identifying concepts of medication literacy: an international perspective. Res Soc Administr Pharm. (2017) 14:797–804. 10.1016/j.sapharm.2017.11.005 29191647

[2] Zheng F Ding S Zhong Z Pan C Xie J Qin C Investigation on status of discharged patients' medication literacy after coronary artery stent implantation. Chin Nurs Res. (2015) 29:1732–4. 10.3969/j.issn.10096493.2015.14.024

[3] Kripalani S Henderson LE Jacobson TA Vaccarino V. Medication use among inner-city patients after hospital discharge: patient-reported barriers and solutions. Mayo Clin Proc. (2008) 83:529–35. 10.4065/83.5.529 18452681

[4] Hu C Wu Q Hu D The status of cardiovascular disease: challenges and strategies. Chin J Hypertens. (2015) 23:625–6.

[5] Hughes GD Aboyade OM Clark BL Puoane TR. The prevalence of traditional herbal medicine use among hypertensives living in South African communities. BMC Compl Altern Med. (2013) 13:1–8. 10.1186/1472-6882-13-38 23414344 PMC3598715

[6] Weiwei C Runlin G Lisheng L Manlu Z Wen W Yongjun W. Outline of the report on cardiovascular diseases in China, 2014. Eur Heart J Suppl. (2016) 18:F2–F11. 10.1093/eurheartj/suw030 28533724

[7] Behnoodrod A Rabbanifar O Pourzargar P Rai A Saadat Z Saadat H Adherence to antihypertensive medications in iranian patients. Int J Hypertens. (2016) 2016:1–7. 10.1155/2016/1508752 PMC4812400 27069676

[8] Pan J Lei T Hu B Li Q. Post-discharge evaluation of medication adherence and knowledge of hypertension among hypertensive stroke patients in northwestern china. Patient Prefer Adherence. (2017) 11:1915–22. 10.2147/PPA.S147605 29200832 PMC5700759

[9] Kim S Shin DW Yun JM Hwang Y Park SK Ko YJ. Medication adherence and the risk of cardiovascular mortality and hospitalization among patients with newly prescribed antihypertensive medications. Hypertension. (2016) 67:506–12. 10.1161/HYPERTENSIONAHA.115.06731 26865198

[10] Peng X Mo XY Ning YY Investigation of irrational drug use in elderly patients with hypertension in outpatient department and nursing countermeasures. Nurs Res. (2013) 27:1561–2.

[11] Maniaci MJ Heckman MG Dawson NL. Functional health literacy and understanding of medications at discharge. Mayo Clin Proc. (2008) 83:520–2. 10.1016/S0025-6196(11)60728-3 18452685

[12] Choi J. Literature review: using pictographs in discharge instructions for older adults with low-literacy skills. J Clin Nurs. (2011) 20:2984–96. 10.1111/j.1365-2702.2011.03814.x 21851434

[13] Herlinda Z Clingerman EM Health literacy among older adults: a systematic literature review. J Gerontol Nurs. (2011) 37:41 10.3928/00989134-20110503-02 21634314

[14] King JL Schommer JC Wirsching RG. Patients' knowledge of medication care plans after hospital discharge. Am J Health-Syst Pharm. (1998) 55:1389. 10.1093/ajhp/55.13.1389 9659968

[15] Hochhalter AK Basu R Prasla K Jo C. Retrospective cohort study of medication adherence and risk for 30-day hospital readmission in a medicare cost plan. Managed Care. (2014) 23:43. 24765750

[16] Sarkar U López A Maselli JH Gonzales R. Adverse drug events in U.S. adult ambulatory medical care. Health Serv Res. (2011) 46:1517–33. 10.1111/j.1475-6773.2011.01269.x 21554271 PMC3168717

[17] Toren O Kerzman HN Baron-Epel O. Patients' knowledge regarding medication therapy and the association with health services utilization. Eur J Cardiovasc Nurs. (2006) 5:311–6. 10.1016/j.ejcnurse.2005.12.001 16427361

[18] Yedidia MJ Gillespie CC Elizabeth K Schwartz MD Judith O Chepaitis AE. Effect of communications training on medical student performance. JAMA. (2003) 290:1157–65. 10.1001/jama.290.9.1157 12952997

[19] Zheng F Ding S Luo A Zhong Z Duan Y Shen Z. Medication literacy status of outpatients in ambulatory care settings in changsha, china. J Int Med Res. (2017) 45:303–9. 10.1177/0300060516676726 28222647 PMC5536586

